# Evaluation of bone mineral density and bone/muscle geometry using pQCT in children after spinal cord injury

**DOI:** 10.1186/1687-9856-2013-S1-O41

**Published:** 2013-10-03

**Authors:** CF Munns, A Biggin, A Middleton, KA Ramjan, JN Briody, MCA Waugh

**Affiliations:** 1 Institute of Endocrinology & Diabetes, The Sydney Children’s Hospitals Network, Sydney, Australia; 2 Discipline of Paediatrics & Child Health, University of Sydney, Sydney, Australia; 3 Department of Nuclear Medicine, The Sydney Children’s Hospitals Network, Sydney, Australia; 4 Department of Rehabilitation Medicine, The Sydney Children’s Hospitals Network, Sydney, Australia

## 

Spinal cord injury (SCI) is associated with a reduction in bone mineral density (BMD) and increasedbone fragility. This study aimed to quantify regional changes in bone mineral density and bone/muscle geometry in children following SCI using Peripheral Quantitative Computer Tomography (pQCT).

A retrospective cohort study of 19 patients (10 males and 9 females) with SCI was undertaken. The group comprised 9 paraplegics (6 complete, 3 incomplete) and 10 tetraplegics (5 complete, 5 incomplete). The mean age at SCI was 6.6 years. pQCT assessment was performed at a mean of 5.7 years post SCI. A total of 7 patients also had serial scans performed: The first at a mean of 7.6 years and the second at a mean of 10.7 years post SCI.

Reduced bone mass following SCI was regional. Lower limb involvement was universal and upper limb involvement was only seen in association with tetraplegia (Table 1). There was a significant loss of muscle cross sectional area in the calves.Analysis of serial pQCT data revealed a further reduction in trabecular volumetric bone mineral density (vBMD) Z-scores between 7.6 and 10.7 years post SCI, while cortical vBMD did not change.Incomplete paraplegics and tetraplegics, who were able to stand, had greater trabecular vBMD, cortical bone mineral content and cortical thickness tibial Z-scores than those with a complete SCI.

**Table 1:** pQCT data of tibial and radial Z-scoresat 4% and 66% sites

	4% site	66% site
Tibial Z-score:		

vBMD trabecular	-2.9 +/- 1.3*	

vBMD cortical		0.5 +/- 1.5

Total CSA		-1.8 +/- 2.6*

BMC		-3.9 +/- 3.4*

pSSI		-2.7 +/- 3.5*

Radial Z-score:		

vBMD trabecular	-3.2 +/- 3.6*	

vBMD cortical		-0.2 +/- 3.1

Total CSA	-1.4 +/- 1.9*	-1.1 +/- 0.8*

BMC	-2.5 +/- 1.5*	-3.9 +/- 3.0*

pSSI		-1.7 +/- 1.5*

Values represent mean Z-scores +/- SD, asterisk represents p<0.05 compared to controls.

Tibial data for paraplegics and tetraplegics (n=19), radial data for tetraplegics only (n=10)

vBMD - volumetric bone mineral density, CSA - cross sectional area, BMC - bone mineral content,

pSSI – polar strength-strain index

pQCT provides a valuable insight into the regional changes in bone and muscle development in children following SCI. Residual muscle function with the ability to weight bear, even if only in a frame, provides a significant benefit to bone development.

